# Extent of the low-density line is associated with time to fracture in children with congenital anterolateral bowing of the tibia: a retrospective survival analysis

**DOI:** 10.3389/fped.2026.1836191

**Published:** 2026-06-15

**Authors:** Jiaqi Tian, Ge Yang, Yonghong Xie, Le Xu, Lan Yin, Yaqi Ouyang, Yinzhi Yi, Jianhui Xie

**Affiliations:** 1 School of Nursing, University of South China, Hengyang, China; 2 Department of Orthopedics, Hunan Children’s Hospital, Changsha, China; 3 Hospital Administration Office, Hunan Children’s Hospital, Changsha, China

**Keywords:** anterolateral bowing of the tibia, congenital pseudarthrosis of the tibia, low-density line, pediatric, survival analysis, tibial fractures

## Abstract

**Introduction:**

Congenital anterolateral bowing of the tibia (ALBT) represents a critical pre-fracture stage in the progression toward congenital pseudarthrosis of the tibia. The low-density line (LDL) is a characteristic radiographic feature, but its temporal relationship with fracture risk remains unclear. This study aimed to evaluate the association between LDL extent and time to fracture and to identify clinically relevant temporal risk patterns.

**Methods:**

In this retrospective cohort study, eligible participants were children diagnosed with ALBT and radiographically confirmed LDL who were treated at Hunan Children’s Hospital between January 2015 and December 2020. LDL extent was quantified as the proportion of the LDL area (*S*_1_) relative to the apparent cross-sectional area of the tibial shaft at the same level (S) on radiographs, calculated as *k* = *S*_1_/*S*, and categorized into three groups (*k* < 1/3, 1/3 ≤ *k* < 2/3, and *k* ≥ 2/3). Time from first LDL detection to fracture or last follow-up was analyzed using Kaplan–Meier survival analysis and Cox proportional hazards models.

**Results:**

A total of 40 children were included. Fractures occurred in 31 (77.5%), with 61.3% of fracture events occurring within 6 months after LDL detection. The Kaplan–Meier-estimated cumulative fracture probability at 6 months was 47.5%. Kaplan–Meier analysis showed significant differences in fracture-free survival among LDL proportion groups (log-rank *χ*^2^ = 8.5, *P* = 0.014), with median times to fracture of 567, 155, and 107 days in the <1/3, 1/3–2/3, >2/3 groups, respectively. On univariable Cox regression, larger LDL proportion was associated with a shorter time to fracture, with significantly higher fracture risk in the 1/3–2/3 group (*HR* *=* 2.784, 95% CI:1.120–6.920, *P* = 0.028) and >2/3 group (*HR* *=* 3.221, 95% CI: 1.324–7.833, *P* = 0.010) compared with the <1/3 group (overall *P* = 0.021). After multivariable adjustment, this association was attenuated and no longer statistically significant (overall *P* = 0.145).

**Conclusion:**

In this LDL-positive pediatric ALBT cohort, LDL proportion may be associated with earlier fracture timing. The clustering of fracture events within the first 6 months after LDL detection may indicate a potential period for closer monitoring. These findings provide preliminary evidence supporting LDL as an imaging marker for time-based risk stratification. Further validation in larger prospective studies is required.

## 1 Introduction

Congenital pseudarthrosis of the tibia (CPT) is one of the most challenging conditions in pediatric orthopedics. The natural course typically follows a staged progression, beginning with congenital anterolateral bowing of the tibia (ALBT) and eventually leading to pathological fracture and pseudarthrosis formation (1–3). ALBT is widely regarded as the early stage of CPT, with an estimated incidence of 1/250,000–1/53,000 (4). Radiographically, ALBT is characterized by anterolateral angulation of the mid-to-distal tibia, often accompanied by cortical thickening, narrowing of the medullary canal, or cystic lesions. Following spontaneous or minor trauma-induced fracture, bone healing is frequently impaired, ultimately resulting in tibial pseudarthrosis (5–7). The Crawford classification describes this radiographic spectrum, with types I–III representing pre-pseudarthrosis stages and type IV indicating established pseudarthrosis (8).

The transition from Crawford types I–III to type IV—that is, from bowing deformity to fracture—represents a critical disease turning point. Once fracture occurs, achieving and maintaining bone union becomes substantially more difficult, and the risk of recurrent fracture and limb-length discrepancy increases considerably (6, 9, 10). A recent case report has also highlighted the clinical heterogeneity of anterolateral congenital tibial bowing and the importance of individualized assessment before fracture onset (11). Therefore, the pre-fracture period in ALBT is a particularly important window for clinical surveillance and timely intervention. However, clinical observations suggest substantial variability in the rate of this progression: some patients maintain cortical continuity for prolonged periods, whereas others develop fractures rapidly. This variability suggests that fracture risk is not uniform and may be influenced by specific radiographic features at presentation. Identifying imaging markers that can predict not only fracture risk but also the likely timing of fracture would therefore be of considerable clinical value. In our previous study, we found that the presence of a low-density line (LDL) on tibial radiographs in patients with non-Crawford type IV CPT was significantly associated with fracture risk, and that a greater LDL proportion relative to the cross-sectional area of the tibial shaft was associated with higher fracture risk (12). This finding introduced LDL as a potential imaging marker in CPT risk assessment. However, the temporal pattern of fracture occurrence following LDL appearance remains unclear, particularly whether different LDL proportions correspond to different fracture risk windows.

Therefore, the present study used survival analysis to investigate the association between quantitative LDL characteristics and time to fracture in children with ALBT, with the aim of clarifying the temporal pattern of fracture risk and providing evidence to guide follow-up monitoring and early intervention.

## 2 Materials and methods

### 2.1 Study design and participants

This was a single-center retrospective cohort study. Patients were identified from the medical records of Hunan Children's Hospital between January 2015 and December 2020.

The inclusion criteria were as follows: (1) diagnosis of non–Crawford type IV CPT; (2) age <18 years at the first hospital visit; (3) complete clinical and radiographic records with a follow-up duration >6 months; (4) radiographic identification of LDL preceding any fracture event.

The exclusion criteria were: (1) pseudarthrosis caused by identifiable acquired factors (e.g., tibial osteomyelitis); (2) concomitant traumatic injury at presentation; (3) initial diagnosis of Crawford type IV CPT; (4) absence of LDL on imaging or appearance of LDL after fracture; (5) follow-up duration <6 months.

### 2.2 Data collection

Clinical data were extracted from medical records and imaging archives. The collected variables included: (1) baseline characteristics: sex, age at first visit, age at symptom onset, affected limb side, presence of neurofibromatosis type 1 (NF1), occurrence of tibial fracture, and history of trauma; (2) radiographic information: Crawford classification and distribution and progression of the low-density line; (3) treatment and follow-up information: surgical or conservative treatment strategies, interventions before and after the appearance of LDL, and follow-up duration.

Data extraction was performed independently by two investigators and subsequently verified by a senior physician. Discrepancies were resolved through discussion.

This study was approved by the Ethics Committee of Hunan Children's Hospital (HCHLL-2021-125). Written informed consent was obtained from the legal guardians of all participants prior to enrollment. The study was conducted in accordance with the Declaration of Helsinki.

### 2.3 Radiographic assessment

Radiographic assessment was performed using the first available tibial radiographs at presentation, including both in-hospital and external imaging records. In-hospital radiographs were retrieved from the hospital's health information system and picture archiving and communication system (PACS). External radiographs were digitized using a high-resolution camera or scanner and archived in the patient's dedicated medical record.

### 2.4 Definition of LDL

**Figure 1:**
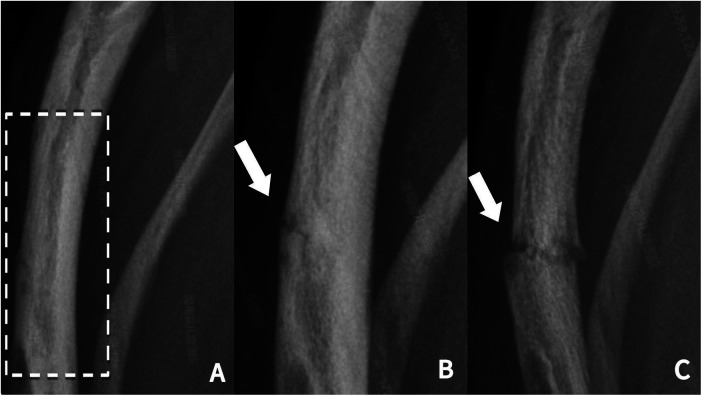
Typical lateral radiographic manifestations of low-density lines in children with ALBT. **(A)** Initial radiograph showing intact cortical continuity with no identifiable LDL. **(B)** Follow-up radiograph demonstrating a linear radiolucent band within the anterior tibial cortex (arrow), consistent with LDL appearance. **(C)** Radiograph obtained approximately 4 months after LDL detection, showing progression to complete tibial fracture at the site of the previously identified LDL (arrow). All three images are lateral views.

The grayscale of the adjacent normal tibial cortex was used as a reference. A linear radiolucent band with significantly lower density than the surrounding bone was defined as an LDL (Figure 1).

**Figure 2:**
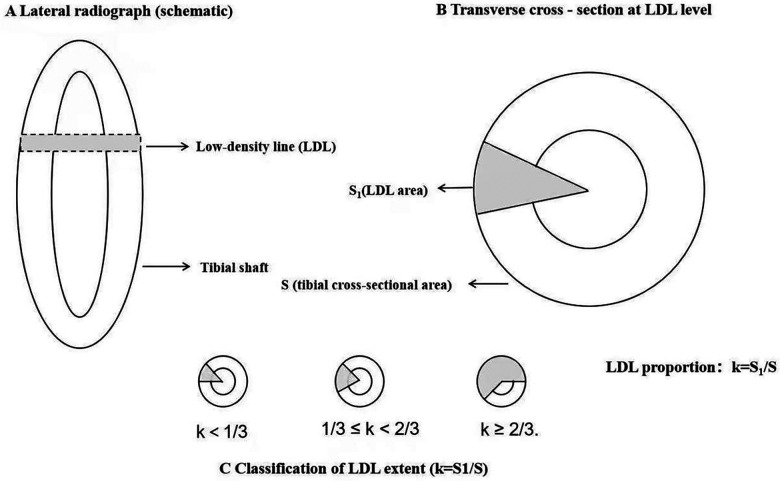
Schematic illustration of low-density line (LDL) measurement and classification. **(A)** Lateral radiograph schematic showing the LDL as a radiolucent band (grey dashed stripe) within the tibial shaft. **(B)** Transverse cross-section at the level of the LDL: S denotes the total tibial cross-sectional area; S₁ denotes the area occupied by the LDL. The proportion coefficient was calculated as *k* = *S*₁/*S*. **(C)** Classification of patients into three groups based on k value: *k* < 1/3, 1/3 ≤ *k* < 2/3, and *k* ≥ 2/3.

Quantitative measurement of LDL:The extent of the LDL was assessed on high-resolution lateral tibial radiographs using image analysis tools embedded in the hospital Picture Archiving and Communication System (PACS). The area of the LDL region was measured (recorded as S_1_), and the cross-sectional area of the tibial shaft at the same level was measured (recorded as S). The proportion coefficient was calculated as *k* = *S*_1_/*S*. Patients were categorized into three groups: *k* < 1/3, 1/3 ≤ *k* < 2/3, and *k* ≥ 2/3 (Figure 2).

Crawford classification:Crawford type I is characterized by cortical thickening with a patent medullary canal at the apex of the deformity. Type II shows medullary narrowing with cortical thickening and trabecular loss. Type III presents with cystic lesions of the tibia. Type IV indicates established tibial pseudarthrosis, sometimes accompanied by fibular pseudarthrosis (8).

### 2.5 Outcome definition and survival time

The primary outcome was the occurrence of a new complete tibial fracture confirmed by clinical symptoms and radiographic findings.

Time-to-event analysis was performed using the first radiographic confirmation of LDL as the observation starting point (t₀). Survival time was defined as follows: for patients who developed fracture, survival time = date of fracture diagnosis − *t*_0_; for patients without fracture, survival time = date of last radiographic follow-up – *t*_0_ (censored observation).

### 2.6 Statistical analysis

Statistical analyses were performed using IBM SPSS Statistics version 27.0. Continuous variables were described as mean ± standard deviation (*x¯* *±* *s*) for normally distributed data or median with interquartile range [*M* (*Q*_1_, *Q*_3_)] for non-normally distributed data. Categorical variables were expressed as frequencies and percentages [*n* (%)]. Given the limited sample size and uneven distribution of some categorical variables, Fisher's exact test was used for group comparisons. Variables with extremely imbalanced distributions or risk of complete separation were described descriptively only.

For survival analysis, the time of first radiographic identification of LDL was defined as the starting point, and the first fracture event was defined as the endpoint. Patients without fracture at the end of follow-up were treated as censored observations. Fracture-free survival curves were estimated using the Kaplan–Meier method, and differences between groups were compared using the log-rank test. The 6-month fracture-free survival probability was estimated from the Kaplan–Meier curve as an exploratory *post hoc* analysis, and the corresponding cumulative fracture probability was calculated as 1 − *S*(180 days). The 6-month time point was used descriptively and was not considered a prespecified prognostic cut-off. A Cox proportional hazards model was used to further explore the association between LDL characteristics and time to fracture. Given the limited sample size and the resultant constraints on statistical power, the Cox regression analysis was conducted as an exploratory analysis rather than a confirmatory one. Covariates were selected based on univariable analysis (*P* < 0.10) and clinical relevance. Model assumptions were evaluated as follows: multicollinearity among covariates was assessed using variance inflation factors (VIF), and the proportional hazards assumption was evaluated using Schoenfeld residuals. Results were reported as hazard ratios (HRs) with 95% confidence intervals (95% CIs). All statistical tests were two-sided, and *P* < 0.05 was considered statistically significant.

## 3 Results

### 3.1 Baseline characteristics

A total of 40 patients were included in the study. During follow-up, 31 patients developed tibial fracture, whereas 9 patients remained fracture-free. The median age at diagnosis was 21 months (*IQR*, 15–37.5 months), and the median follow-up duration was 68.8 months (*IQR*, 45.4–108.6 months).

Baseline demographic and clinical characteristics are presented in Table 1. No statistically significant difference was observed in sex distribution between the fracture and non-fracture groups (*P* = 0.052). No significant differences were found in age at first LDL detection, presence of neurofibromatosis type 1 (NF1), or Crawford classification between the two groups (all *P* > 0.05).

**Table 1:** Baseline demographic and clinical characteristics of children with anterolateral bowing of the tibia (ALBT) and radiographically confirmed low-density line (LDL) (*n* = 40).

Characteristic	Total (*N* = 40)	Fracture group (*n* = 31)	Non-fracture group (*n* = 9)	*P* value
Sex				0.052
Male	22 (55.0)	14 (45.2)	8 (88.9)	
Female	18 (45.0)	17 (54.8)	1 (11.1)	
Crawford classification (8)				0.890
Type I	7 (17.5)	5 (16.1)	2 (22.2)	
Type II	20 (50.0)	16 (51.6)	4 (44.4)	
Type III	13 (32.5)	10 (32.3)	3 (33.3)	
NF1 status (27)				0.170
NF1-positive	31 (77.5)	26 (83.9)	5 (55.6)	
NF1-negative	9 (22.5)	5 (16.1)	4 (44.4)	
Affected limb				—
Left	21 (52.5)	17 (54.8)	4 (44.4)	
Right	17 (42.5)	14 (45.2)	3 (33.3)	
Bilateral	2 (5.0)	0 (0)	2 (22.2)	
Prior surgical history				—
No	1 (2.5)	1 (3.2)	0 (0)	
Yes	39 (97.5)	30 (96.8)	9 (100)	
Staple use				1.000
No	27 (67.5)	21 (67.7)	6 (66.7)	
Yes	13 (32.5)	10 (32.3)	3 (33.3)	

Data are presented as *n* (%). Statistical comparisons were performed using Fisher’s exact test. “—” indicates that statistical testing was not applicable or not performed. *P* < 0.05 was considered statistically significant. NF1, neurofibromatosis type 1.

### 3.2 Distribution of LDL radiographic characteristics

According to the proportion of LDL within the cross-sectional area of the tibial shaft, 47.5% of patients were classified into the <1/3 group, 27.5% into the 1/3–2/3 group, and 25.0% into the >2/3 group. The distribution of LDL proportion, morphology, and anatomical location showed descriptive differences between patients with and without fracture. Detailed information is shown in Table 2.

**Table 2:** Radiographic characteristics of the low-density line (LDL) and fracture status in children with anterolateral bowing of the tibia (*n* = 40).

Characteristic	Total (*N* = 40)	Fracture group (*n* = 31)	Non-fracture group (*n* = 9)	*P* value
LDL proportion within tibial shaft cross-sectional area				0.021
<1/3	19 (47.5)	11 (35.5)	8 (88.9)	
1/3–2/3	11 (27.5)	10 (32.3)	1 (11.1)	
>2/3	10 (25.0)	10 (32.3)	0 (0.0)	
LDL morphology				0.570
Transverse	20 (50.0)	14 (45.2)	6 (66.7)	
Oblique	19 (47.5)	16 (51.6)	3 (33.3)	
Curved	1 (2.5)	1 (3.2)	0 (0)	
LDL anatomical location				0.650
Proximal shaft	2 (5.0)	2 (6.5)	0 (0)	
At the prior surgical site	29 (72.5)	21 (67.7)	8 (88.9)	
Distal shaft	9 (22.5)	8 (25.8)	1 (11.1)	
Fixation status at LDL detection				0.660
No internal or external fixation	36 (90.0)	28 (90.3)	8 (88.9)	
Intramedullary rod fixation	2 (5.0)	2 (6.5)	0 (0)	
Distal screw fixation	2 (5.0)	1 (3.2)	1 (11.1)	
Management after LDL detection				—
No intervention	2 (5.0)	0 (0)	2 (22.2)	
Cast immobilization	10 (25.0)	9 (29.0)	1 (11.1)	
Combined surgical treatment	22 (55.0)	18 (58.1)	4 (44.4)	
Continued internal fixation	1 (2.5)	1 (3.2)	0 (0)	
Brace immobilization	1 (2.5)	1 (3.2)	0 (0)	
Intramedullary rod combined with cast	4 (10.0)	2 (6.5)	2 (22.2)	

Data are presented as *n* (%). Statistical comparisons were performed using Fisher’s exact test. “—” indicates that statistical testing was not applicable or not performed. *P* < 0.05 was considered statistically significant. NF1, neurofibromatosis type 1.

### 3.3 Survival analysis of fracture occurrence after LDL detection

Among the 31 patients who developed fractures, 19 patients (61.3%) experienced fracture within 6 months after LDL detection, 4 patients (12.9%) between 6 and 12 months, and 8 patients (25.8%) after 12 months. In an exploratory *post hoc* Kaplan–Meier assessment of the entire cohort, the estimated fracture-free survival probability at 6 months after LDL detection was 52.5% (SE = 7.9%), corresponding to a cumulative fracture probability of 47.5%.

**Figure 3:**
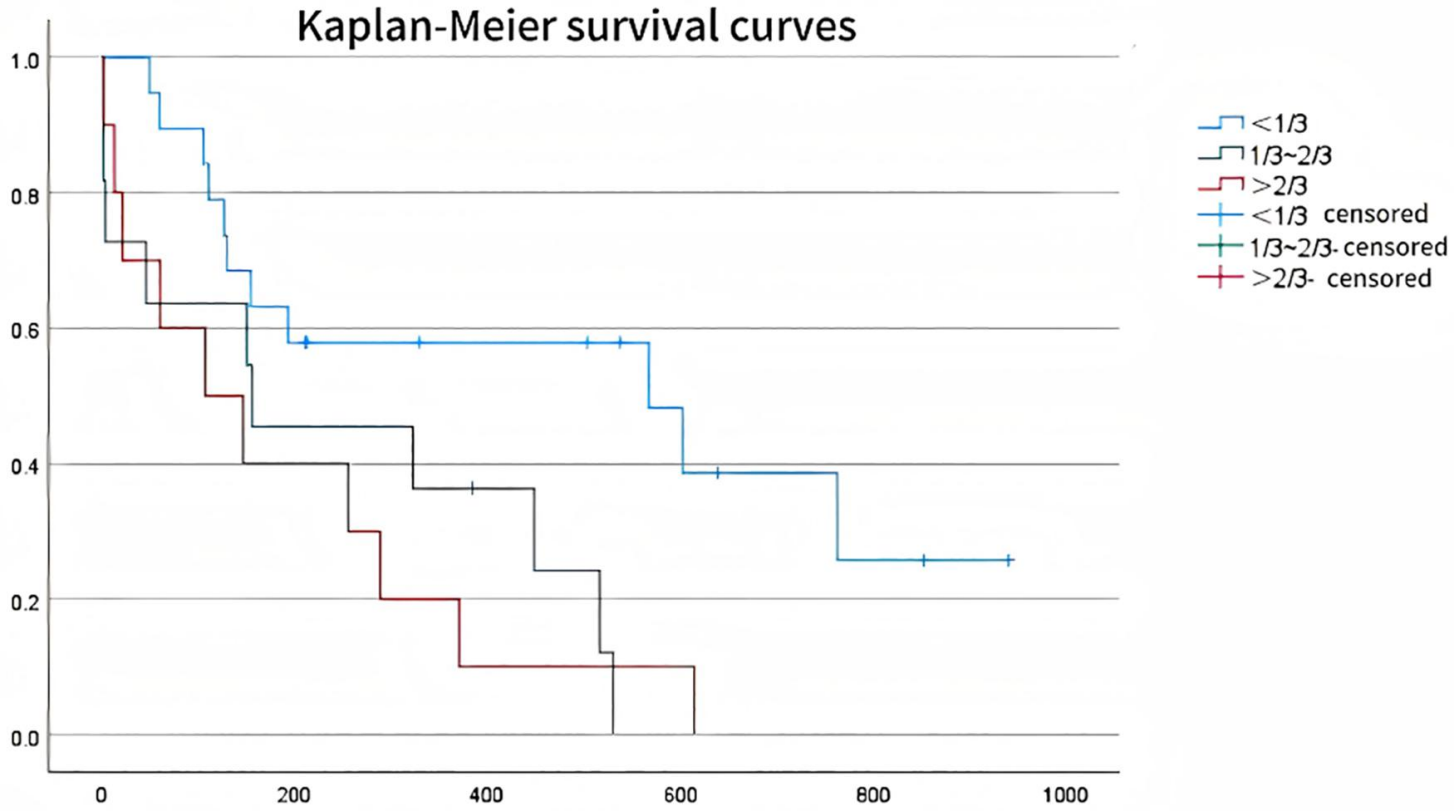
Kaplan–Meier fracture-free survival curves stratified by the proportion of LDL in the tibial diaphyseal transverse diameter. Cumulative fracture-free survival rates are shown for three groups: LDL proportion <1/3 (blue line), 1/3–2/3 (green line), and >2/3 (red line). Censored cases are marked with tick marks. The *x*-axis represents time from first identification of LDL to fracture occurrence (days); the *y*-axis represents cumulative fracture-free survival rate.

Among patients who developed fractures, the median observed time from LDL detection to fracture was 146 days *(IQR*, 49–370 days). Kaplan–Meier analysis demonstrated a significant difference in fracture-free survival among the LDL proportion groups (log-rank test: *χ*^2^ = 8.5, *P* = 0.014). The median time to fracture was 567 days in the <1/3 group, 155 days in the 1/3–2/3 group, and 107 days in the >2/3 group (Figure 3). Censored observations were present in all three groups, with 8, 1, and 0 censored patients in the <1/3, 1/3–2/3, and >2/3 groups, respectively.

Pairwise comparisons showed that the fracture-free survival rate in the <1/3 group was higher than that in the 1/3–2/3 group (*P* = 0.017) and the >2/3 group (*P* = 0.012), whereas no significant difference was observed between the 1/3–2/3 group and the >2/3 group (*P* = 0.666).

### 3.4 Management strategies after LDL detection and fracture outcomes

Among the 40 patients with LDL, the initial management strategies included no specific intervention in 2 patients (5.0%), plaster immobilization in 10 patients (25.0%), combined surgical treatment in 22 patients (55.0%), continued internal fixation in 1 patient (2.5%), brace immobilization in 1 patient (2.5%), and intramedullary rod combined with plaster fixation in 4 patients (10.0%). The detailed treatment strategies and clinical outcomes are presented in Table 3. As treatment strategies were not randomly assigned and reflected clinical decision-making based on disease severity and individual patient factors, no formal statistical comparisons were performed.

**Table 3:** Management strategies following low-density line (LDL) detection and subsequent fracture outcomes in children with anterolateral bowing of the tibia (*n* = 40).

Management strategy	*n*	Fracture	LDL resolution	Incomplete healing with healing tendency	Cortical crack	Enlargement without crack
No intervention	2	0	1	0	0	1
Cast immobilization	10	9	0	0	0	1
Combined surgical treatment	22	18	0	0	3	1
Continued internal fixation	1	1	0	0	0	0
Brace immobilization	1	1	0	0	0	0
Intramedullary rod + cast	4	2	0	0	2	0
Total	**40**	**31**	**1**	**0**	**5**	**3**

This table presents descriptive data only. No formal statistical comparisons were performed between management groups, as treatment strategies were not randomly assigned and reflected clinical decision-making based on disease severity and individual patient factors. “Cortical crack” refers to an incomplete fracture with one cortex remaining intact. “Enlargement without crack” refers to an increase in LDL extent without cortical disruption.

### 3.5 Cox proportional hazards regression analysis

#### 3.5.1 Univariable Cox regression analysis

Univariable Cox regression analysis showed that the proportion of LDL within the cross-sectional area of the tibial shaft was associated with time to fracture (overall *P* = 0.021) (Table 4). Using the <1/3 group as the reference, both the 1/3–2/3 group (*HR* = 2.784, 95% CI: 1.120–6.920, *P* = 0.028) and the >2/3 group (*HR* = 3.221, 95% CI: 1.324–7.833, *P* = 0.010) showed an increased hazard of fracture. Sex was also associated with time to fracture, with male patients showing a lower hazard compared with females (*HR* = 0.199, 95% CI: 0.082–0.481, *P* < 0.001). Age showed a modest negative association with fracture risk, with each additional month associated with a slight reduction in hazard (*HR* = 0.985, 95% CI: 0.971–0.999, *P* = 0.039). NF1 status and Crawford classification were not significantly associated with time to fracture (*P* = 0.259 and *P* = 0.214, respectively).

**Table 4:** Univariable Cox proportional hazards analysis of factors associated with time to tibial fracture in children with anterolateral bowing of the tibia (*n* = 40).

Variable	Coefficient	SE	Wald z	*P* value	HR	95% CI
LDL proportion—<1/3 (reference)	—	—	—	0.021	—	—
1/3–2/3	1.024	0.465	2.217	0.028	2.784	1.120–6.920
>2/3	1.170	0.453	2.586	0.010	3.221	1.324–7.833
Sex—Female (reference)	—	—	—	—	—	—
Male	−1.614	0.450	−3.030	<0.001	0.199	0.082–0.481
Age (months)	−0.015	0.007	−2.068	0.039	0.985	0.971–0.999
NF1 status—NF1-negative (reference)	—	—	—	—	—	—
NF1-positive	0.554	0.491	1.130	0.259	1.739	0.665–4.551
Crawford classification—Type I (reference)	—	—	—	0.214	—	—
Type II	0.800	0.552	1.530	0.125	2.226	0.801–6.189
Type III	0.276	0.559	0.490	0.622	1.317	0.441–3.937

SE, standard error; HR, hazard ratio; CI, confidence interval. LDL, low-density line; NF1, neurofibromatosis type 1.

#### 3.5.2 Multivariable Cox regression analysis

The proportional hazards assumption was satisfied for all covariates based on Schoenfeld residual testing (all *P* > 0.05). No significant multicollinearity was detected among covariates (all *VIF* < 2). Variables including age, sex, and LDL proportion were entered into the multivariable Cox proportional hazards model (Table 5). The overall model was statistically significant (*χ*^2^ = 21.996, *df* = 4, *P* < 0.001). After adjustment for age and sex, the association between LDL proportion and time to fracture was attenuated and did not reach statistical significance (overall *P* = 0.145). Using the <1/3 group as the reference, the >2/3 group showed an increased fracture risk that approached but did not reach statistical significance (*HR* = 2.523, 95% CI: 0.983–6.477, *P* = 0.054). Sex remained associated with time to fracture in the multivariable model (*HR* = 0.308, 95% CI: 0.123–0.771, *P* = 0.012).

**Table 5:** Multivariable Cox proportional hazards analysis of factors associated with time to tibial fracture in children with anterolateral bowing of the tibia (*n* = 40).

Variable	Coefficient	SE	Wald *z*	*P* value	HR	95% CI
LDL proportion—<1/3 (reference)	—	—	—	0.145	—	—
1/3–2/3	0.665	0.486	1.368	0.171	1.945	0.750–5.048
>2/3	0.925	0.481	1.923	0.054	2.523	0.983–6.477
Sex—Female (reference)	—	—	—	—	—	—
Male	−1.179	0.469	−2.515	0.012	0.308	0.123–0.771
Age (months)	−0.013	0.008	−1.584	0.113	0.987	0.970–1.003

SE, standard error; HR, hazard ratio; CI, confidence interval. LDL, low-density line. Overall model: *χ*^2^ = 21.996, *df* = 4, *P* < 0.001. The proportional hazards assumption was verified for all covariates using Schoenfeld residuals (all *P* > 0.05).

## 4 Discussion

ALBT is considered an important clinical stage preceding fracture and pseudarthrosis formation in CPT (13). In this retrospective cohort study, we analyzed the time to fracture following the appearance of LDL in children with ALBT. The results suggested early temporal clustering of fracture events after LDL detection. However, because the 6-month time point was not prespecified, this finding should be interpreted as an exploratory observation indicating a possible period for closer monitoring. Univariable and survival analyses indicated that the proportion of the LDL area relative to the cross-sectional area of the tibial diaphysis was associated with fracture timing, with larger proportions corresponding to earlier fracture occurrence. However, this association did not reach statistical significance after multivariable adjustment. This phenomenon is relatively common in small retrospective studies and should be interpreted with caution (14). Notably, in the group with an LDL proportion >2/3, the adjusted hazard ratio remained 2.523 (95% CI: 0.983–6.477; *P* = 0.054), with a consistent direction of effect, suggesting that the observed association may be relatively robust. The loss of statistical significance is more likely to reflect limited statistical power due to the small sample size rather than the absence of a true effect. Therefore, this study supports interpreting LDL proportion as an imaging risk signal associated with fracture timing rather than establishing it as an independent predictive factor.

Radiographic low-density regions are generally considered to reflect an imbalance between local bone formation and resorption. Such regions exhibit reduced mechanical strength and are prone to stress concentration, which may increase susceptibility to fracture (15–17). In CPT, LDL may represent the radiographic manifestation of this locally compromised structural integrity (11). Histological studies have demonstrated hamartoma-like periosteal proliferation, reduced osteoblastic activity, increased osteoclastic activity, and narrowed, tortuous vascular structures in the affected region, collectively impairing local bone development and promoting bone resorption (18, 19). At the molecular level, loss-of-function alterations in the NF1 gene lead to sustained hyperactivation of the Ras-MAPK (ERK) signaling pathway, which disrupts the balance between osteoblast and osteoclast activity and contributes to the formation of low-density regions (17, 20). A larger LDL proportion therefore reflects a more extensive area of this pathological imbalance, generating broader stress risers and accelerating microdamage accumulation under repetitive physiological loading. This mechanistic cascade may help explain the observed association between LDL extent and earlier fracture timing. LDL likely reflects a localized biomechanical and biological vulnerability that may accelerate fracture occurrence over time.

In this study, NF1 status was not significantly associated with fracture timing. Although germline or somatic NF1 alterations are known to drive the molecular pathology leading to LDL formation, clinical diagnosis of NF1 was not associated with fracture timing in this cohort, consistent with several previous studies showing similar bone pathology and outcomes regardless of systemic NF1 status (21). This finding may be related to the limited sample size and the high proportion of NF1-positive cases, which may have reduced statistical power for between-group comparisons. The role of NF1 in determining fracture timing therefore remains uncertain and warrants further investigation, particularly given the inconsistent findings reported in previous studies regarding its impact on union rates and refracture risk (22, 23). NF1-related molecular alterations may contribute to fracture risk primarily through their effect on LDL formation and extent, rather than independently determining fracture timing once LDL has developed. In this sense, LDL proportion may serve as a more proximal radiographic mediator of fracture timing than NF1 clinical status itself.

The Kaplan–Meier analysis further demonstrated a marked difference in fracture timing across LDL proportion groups. Patients with a larger LDL proportion showed a substantially shorter median time to fracture. This finding suggests that the affected bone may enter a mechanically vulnerable state earlier, potentially leading to accelerated accumulation of microdamage under repetitive physiological loading, although the specific structural alterations within LDL regions remain to be clarified through advanced imaging or histological correlation (24).

From a clinical perspective, the early clustering of fracture events within 6 months after LDL detection suggests a potentially important period for closer monitoring. Closer radiographic follow-up, combined with quantitative assessment of LDL proportion, may enable risk stratification and earlier consideration of protective interventions—such as prolonged bracing or prophylactic surgery—in high-risk cases. LDL extent should nevertheless be interpreted as an adjunctive imaging marker rather than the sole basis for clinical decision-making, as individualized management before fracture onset may also depend on clinical symptoms, functional limitation, and deformity progression (11). However, given the exploratory nature of the present study, specific management strategies and follow-up intervals should be interpreted with caution and require validation in prospective studies.

LDL may not represent a static radiographic feature, but rather a dynamic process reflecting ongoing changes in local bone metabolism and mechanical adaptation. Its extent may evolve over time under the combined influence of biological activity, mechanical loading, and clinical management. Over time, local stability may improve through bone remodeling or clinical intervention, potentially contributing to a reduction in fracture hazard beyond the early follow-up period. In our cohort, most patients received cast immobilization or surgical treatment after LDL detection; however, these interventions were not randomly assigned and were subject to indication bias. Therefore, this study does not attempt to compare treatment effects.

Several limitations should be acknowledged. First, this was a single-center retrospective study with a relatively small sample size, which may have limited statistical power and increased the risk of type II error (25). Second, the potential for selection bias should be acknowledged. Because this study included only patients with radiographically documented LDL before fracture or before the last follow-up, patients who fractured without a visible or documented LDL could not be evaluated. This may limit the generalizability of our findings to the broader ALBT/CPT population, particularly LDL-negative patients. Future prospective studies including both LDL-positive and LDL-negative patients are needed to validate the role of LDL extent in fracture risk stratification. Third, the multivariable Cox regression results should be interpreted as exploratory rather than confirmatory. Although some variables showed potential associations in univariable analysis, these associations were attenuated in the multivariable model, likely reflecting limited statistical power in this small cohort. Finally, the proportion of males in the non-fracture group (88.9%) was notably higher than that in the fracture group (45.2%), suggesting that the observed sex difference may partly reflect imbalance in disease severity rather than an independent biological effect of sex itself (26). Despite these limitations, the consistent direction of the Kaplan–Meier and univariable Cox findings supports LDL extent as a potential imaging marker associated with fracture timing in LDL-positive ALBT patients.

## 5 Conclusion

In this LDL-positive pediatric ALBT cohort, survival and univariable analyses suggest that LDL proportion may be associated with earlier fracture timing. The clustering of fracture events within the first 6 months after LDL detection may indicate a potential period for closer monitoring. These findings indicate that LDL extent may serve as a time-related imaging marker for temporal risk stratification rather than an definitive independent predictor. Larger prospective studies including both LDL-positive and LDL-negative patients are needed to validate these findings.

## Data Availability

The original contributions presented in the study are included in the article/Supplementary Material, further inquiries can be directed to the corresponding author.
